# COVID-19 Vaccines: An Updated Overview of Different Platforms

**DOI:** 10.3390/bioengineering9110714

**Published:** 2022-11-19

**Authors:** Dmitry Kudlay, Andrey Svistunov, Oleg Satyshev

**Affiliations:** 1 Department of Pharmacology, Institute of Pharmacy, I.M. Sechenov First Moscow State Medical University (Sechenov University), 119991 Moscow, Russia; 2 Laboratory of Psychiatric Neurobiology, Institute of Molecular Medicine and Department of Normal Physiology, I.M. Sechenov First Moscow State Medical University (Sechenov University), 119991 Moscow, Russia; 3 JSC Generium, 123112 Moscow, Russia

**Keywords:** immunization, prevention, vector, mRNA, protein subunits, COVID-19, booster, Omicron

## Abstract

Vaccination has been identified as a critical method of disease control in the context of the current COVID-19 pandemic. The goal of this review is to update information on vaccine development and to identify areas of concern that require further research. We reviewed the literature on the development of COVID-19 vaccines, their efficacy, and use in special populations, as well as current vaccination strategies. To date, 170 vaccines are in clinical development, with 41 being already approved for use in various countries. The majority of vaccines approved for human use are vector-, subunit-, DNA-, or mRNA-based vaccines, or inactivated viruses. Because of the ongoing mutation of the SARS-CoV-2 virus, well-studied vector vaccines are losing relevance due to the ability of new virus strains to bypass neutralizing antibodies. Simultaneously, PS-based vaccines are becoming more popular. There is mounting evidence that the immunogenicity of COVID-19 vaccines is linked to their clinical efficacy. This has resulted in a shift in vaccination strategies, as well as the use of booster doses and revaccination. Furthermore, vaccination restrictions for children, pregnant women, the elderly, and people with chronic immunosuppressive diseases have been lifted, allowing more people to be vaccinated. New data on vaccine safety, including the incidence of serious adverse events, have been collected. Despite significant advances in the development of and research on COVID-19 vaccines, many questions remain that require further investigation.

## 1. Introduction

According to the World Health Organization (WHO), more than 628 million confirmed cases of coronavirus disease 2019 (COVID-19) and approximately 6.5 million COVID-19 deaths had been reported worldwide as of 1 November 2022 [1].

Various COVID-19 treatment strategies, including antiviral and immune therapy, have been developed and implemented during the pandemic [2]. However, vaccination is the primary method of combating the pandemic, with the goal of reducing hospitalizations, the proportion of severe COVID-19, and COVID-19 mortality. Because the SARS-CoV-2 virus has evolved significantly, vaccines must now develop a broader spectrum of immune defense mechanisms that target both currently circulating strains and new variants of the virus. The Technical Advisory Group on COVID-19 Vaccine Composition suggests including SARS-CoV-2 Omicron variant antigens in vaccine booster doses [3].

COVID-19 vaccines had been administered in a total of 12,449,443,718 doses as of 30 August 2022 [1].

Approximately 67.6% of the world’s population has received at least one dose of the vaccine, with 5.1 million doses being administered daily. Portugal and Cuba rank first in terms of the percentage of fully vaccinated people (95% each). The vaccination rate in low-income countries is only 20.9% [4].

As of 30 August 2022, Gibraltar (355.75 vaccine doses per 100 people), Chile (321.87 vaccine doses per 100 people), and Cuba (328.82 vaccine doses per 100 people) ranked first in terms of vaccine doses per person. According to WHO statistics, Gibraltar had 107 COVID-19 deaths; Chile had 60,435; and Cuba had 8530 [5].

The vaccination of children and adolescents, the elderly, and pregnant women becomes increasingly important as time passes, as do determining the most effective vaccines and monitoring their efficacy to keep up with new virus mutations. This review discusses existing vaccine platforms and the main data available on the clinical use of vaccines in order to prioritize research.

This is an update to our previous review, which was published in December 2021 [6].

## 2. COVID-19 Vaccine Platforms and Their Characteristics

As of 30 August 2022, the WHO reported that 198 vaccines were in preclinical trials and 170 vaccines were in clinical trials. Forty-five vaccines are currently in phase 3 clinical trials, with eleven being already in phase 4. In total, 23% are single-dose vaccines, while 56% are double-dose vaccines, and 1% are triple-dose vaccines. In total, 3% of vaccines are intended for oral administration, 8% for subcutaneous and intradermal administration, 7% for intranasal administration, and 1% for use as aerosols or inhalers. The intramuscular route is the most commonly used (82%) for vaccine administration [7].

**Figure 1:**
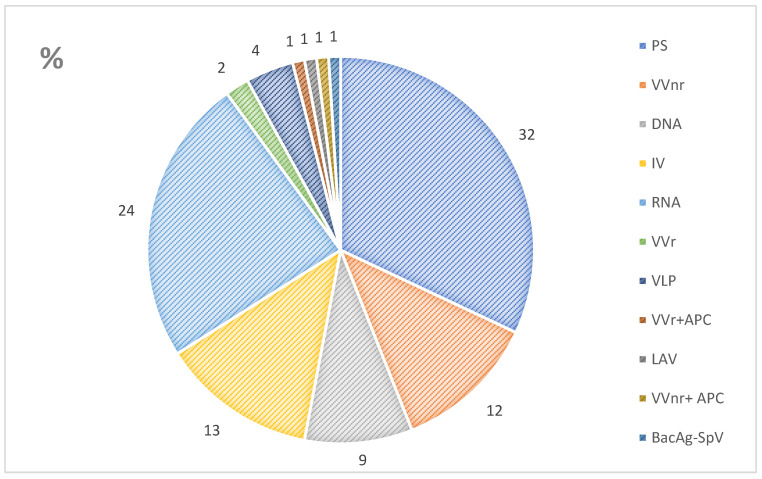
Proportion of vaccine platforms in clinical development [7]. Notes: PS, protein subunit; VVnr, viral vector non-replication; DNA, deoxyribonucleic acid; IV, inactivated virus; RNA, ribonucleic acid; VVr, viral vector replicating; VLP, virus-like particle; APC, antigen presentation cell; LAV, live attenuated virus; BacAg-SpV, bacterial antigen-spore expression vector.

This review focuses solely on vaccines in clinical development. Figure 1 shows vaccine development platforms [7]. The most common vaccines in development are protein subunit (PS) vaccines, followed by ribonucleic acid (RNA) vaccines. These are followed by inactivated virus (IV) and non-replicating viral vector (VVnr) vaccines. Furthermore, replicating viral vector (VVr), virus-like particle (VLP), live attenuated virus (LAV), and bacterial antigen-spore expression vector (BacAg-SpV) vaccines are being developed (Figure 1) [7].

The highest numbers of vaccines are used in Iran (12 vaccines) and in the Philippines, India, and Indonesia (11 vaccines each), and the highest numbers of vaccine trials are currently underway in the United States (104 vaccines), China (98 vaccines), and India (36 vaccines). A total of 6 vaccines have been approved in Russia, and 33 are being studied (11 in phase 1 trials, 14 in phase 2 trials, and 8 in phase 3 trials) (Table 1) [8].

### 2.1. Protein Subunit (PS) Vaccines

This is the primary platform for many vaccines, both approved and in development [9]. Protein subunits are responsible for the formation of neutralizing antibodies as well as the activation of T cells [10]. PS-based vaccines are gaining popularity due to their ability to elicit stronger immune responses based on neutralizing antibodies using only immunodominant epitopes rather than larger viral proteins or whole inactivated virus [11]. The absence of serious side effects is one of the benefits of vaccines on this platform [12]. COVID-19 vaccine protein subunits do not contain live viruses; thus, they cannot cause infection [13]. Disadvantages include the need for revaccination, as well as the use of booster doses to maintain a stronger and longer immune response. Another disadvantage is that the vaccine’s effect is dependent on the adjuvant [12].

The entire S protein (BNT162b2/COMIRNATY Tozinameran (INN) (Pfizer, USA (BNT162b2)); NVX-CoV2373/Covovax/Nuvaxovid/Novavax (Novavax, India (NVX-CoV2373)); MVC-COV1901 (Medigen, Germany (MVC-COV1901)); mRNA-1273/Spikevax (Moderna, USA (mRNA-1273)); and EpiVacCorona (Vector State Research Center of Virology and Biotechnology, Russia)), its receptor-binding domain (RBD) (Abdala, CIGB, Cuba (Abdala)), which is responsible for the binding of the virus to the angiotensin-converting enzyme 2 (ACE) receptor, and the N-terminal domain (NTD) are primarily used in the development of COVID-19 vaccines [14,15].

According to the WHO, there were 22 protein subunit vaccines in phase 2/3–3 clinical trials and 1 in phase 4 clinical trials as of August 2022. The majority of existing vaccines were developed on this platform [9].

Several peptide-based vaccines are currently in phase 3 clinical trials, e.g., SARS-CoV-2 rS/Matrix M1-Adjuvant, Novavax, Sweden, Abdala, and EpiVacCorona [11]. However, EpiVacCorona production in Russia was halted in 2022, and the production of the AURORA-CoV/EpiVacCorona-N vaccine began (Vector State Research Center of Virology and Biotechnology, Russia (AURORA-CoV)) [16,17].

### 2.2. Vaccines Containing Non-Replicating Viral Vector (VVnr)

VVnr vaccines contain SARS-CoV-2 genetic material encapsulated in a viral (vector) envelope; the vector can enter human cells but cannot replicate there. Vectors include adenoviruses, measles, smallpox, and vesicular stomatitis viruses. The advantage of such vaccines is that they produce long-lasting immunity. Because of the mucosal tropism of the virus, adenovirus vector-based vaccines can be administered intranasally [18].

At the time of this review, all registered VVnr vaccines were based on one or more of the adenoviral vectors listed below:

- Chimpanzee adenovirus vector ChAdOx1 (formerly known as ChAdY25). The AZD1222/Vaxzevria/Covishield vaccine (AstraZeneca, UK (AZD1222)) is based on this vector [17];
- Recombinant human adenovirus type 26 (Ad26) vector, contained in the Ad26.COV2.S/Jcovden (Janssen Pharmaceutical/Johnson & Johnson, Belgium/the Netherlands (Ad26.COV2.S)). It is recommended for people over the age of 18 who do not have access to other vaccines [18,19]. The same vector is used in Sputnik Light, (Gamaleya National Center of Epidemiology and Microbiology, Russia (Sputnik Light)). The Sputnik Light vaccine has the advantage of forming strong immunity after a single injection, can be used as a booster, and is effective against the Delta and Omicron strains [20];
- The Ad5-nCoV vaccine/Convidicea (CanSino Biologics, China (Ad5-nCoV)) is based on an adenovirus type 5 (Ad5) vector [21];
- The Sputnik V/Gam-COVID-Vac vaccine (Gamaleya National Center of Epidemiology and Microbiology, Russia (Sputnik V)) contains Ad26 and Ad5 vectors [18,20].

Except for Ad26.COV2.S and Sputnik Light, which are given only once, all the vaccines mentioned above are given twice (i.e., two doses of vaccine are required for full vaccination). All vaccine vectors contain a viral RNA fragment with the viral spike (S) protein, which mediates virus binding and translocation into the cell. As a result, the SARS-CoV-2 antigen is synthesized in human cells following vaccination [22]. Even a single injection of VVnr vaccine is immunogenic enough to produce protective antibody titers [23]. This enables the development and approval of single-phase vaccines. The main issue with VVnr is the retention of immunogenicity after repeated administration; the solution was found in vector alternation and the use of pegylated forms [24]. The effect of polyethylene glycol (PEG) on post-vaccination immunity is debatable. Recent research has found that anti-PEG antibodies have no effect on the specific neutralizing response of anti-SARS-CoV-2 antibodies after vaccination, but that high levels of anti-PEG vaccine-induced antibodies correlate with increased systemic reactogenicity after two doses of the vaccine [25,26].

### 2.3. DNA Vaccines

Nucleic acid vaccines work by introducing genetic material encoding immunogenic virus fragments into human cells. Following the delivery of the genetic material (RNA or deoxyribonucleic acid (DNA)), viral proteins are synthesized, which initiates the immune response and the production of antibodies against the virus. When DNA vaccines are used, the DNA is transcribed into messenger RNA upon reaching the host cells, which is then followed by protein synthesis [27]. DNA vaccines stimulate CD8 and CD4 T cells, eliciting both humoral and cellular immune responses [11]. DNA vaccines have several advantages, including rapid development and exceptional safety [28]. The disadvantages of DNA vaccines over RNA vaccines include the need for additional stages in the formation of an immune response [29]. Two vaccines are currently registered: NO-4800 (Inovio Pharmaceuticals, Plymouth, PA, USA (NO-4800) [21]) and ZyCoV-D (Zydus Cadila, Ahmedabad, India (ZyCoV-D) [9]).

### 2.4. Vaccines Containing Inactivated Virus

Vaccines on this platform are popular due to their quick ability to adjust vaccine composition when the virus strain mutates, as well as their ease of production in comparison with other types of vaccines. Furthermore, these vaccines are simple to mass-produce. The disadvantage of this platform is the possibility of infection spreading due to insufficient pathogen inactivation [30]. Another significant disadvantage of inactivated vaccines is that they may be less immunogenic than mRNA or vector vaccines [31]. The WHO recommends using this type of COVID-19 vaccine in the elderly, healthcare workers, and immunocompromised patients [32].

The following vaccines are already approved and in use: COVID-19 Vaccine (Vero Cell), Inactivated/Coronavac^TM^ (Sinovac, China (COVID-19 Vaccine (Vero Cell), Inactivated)); BBIBP-CorV/SARS-CoV-2 Vaccine (Vero Cell), Inactivated (lnCoV)/Covilo (Sinopharm/BIBP, China (BBIBP-CorV)); BBV152/SARS-CoV-2 Vaccine, Inactivated (Vero Cell)/COVAXIN (Bharat Biotech, India (BBV152)); KoviVac (Chumakov Federal Scientific Center for the Research and Development of Immunobiologicals, Russia (KoviVac)). Two doses of these vaccines are required, which is one of their distinguishing features [21]. Other examples include Turkovac (Health Institutes of Turkey, Turkey (Turkovac)), FAKHRAVAC/MIVAC (Organization of Defensive Innovation and Research, Iran (FAKHRAVAC)), QazVac/QazCovid-in (Research Institute for Biological Safety Problems (RIBSP), Kazakhstan (QazVac)), KCONVAC (Shenzhen Kangtai Biological Products Co, China (KCONVAC)), and COVIran Barekat (Shifa Pharmed Industrial Co, Iran (COVIran Barekat)). All recent vaccines have only been approved in 1–2 countries and are only available in those countries [9].

The vaccine on this platform, COVID-19 Vaccine (Vero Cell), Inactivated, is more effective after two doses than BNT162b2 in reducing hospitalizations by 33.1% (95% CI, 14.5–47.7), intensive care admissions by 47.2% (95% CI, 18.5–65.8), and mortality by 55.7% (95% CI, 32.5–70.0) [33].

### 2.5. RNA Vaccines

Unlike DNA vaccines, the use of an mRNA-based platform eliminates the stage of RNA transcription in human cells, which speeds up and simplifies antigen synthesis [27]. Because the alphavirus nsP1-4 protein is included in the original RNA or is present as a separate fragment, mRNA vaccines can self-actualize. The vaccine’s self-amplification results in a stronger immune response, allowing lower doses to be used [34]. This vaccine induces both cell-mediated and humoral immune responses [10].

RNA vaccines already approved by the WHO include mRNA-1273 and BNT162b2, [21], as well as TAK-919 (Moderna formulation) (has the same formula as mRNA-1273), registered only in Japan. BNT162b2 has the highest number of countries that have approved the vaccine (146 countries) [9].

### 2.6. Viral Vector (Replicating)

There are currently no registered vaccines on this platform. Two oral vaccines are currently being developed: Oral Salmonella-Based Vaccine Platform (Korea), based on Salmonella expressing a modified spike protein [35]; and B. subtilis oral vaccine (DreamTec Research Limited, Hongkong, China), expressing sRBD on the spore surface [36].

### 2.7. Pathogen-Specific aAPCs

APC-based vaccines contain laboratory dendritic cells (DCs) that have the viral antigen on their surface. SARS-CoV-2 causes DC dysfunction, making DCs a viable vaccine target. Furthermore, DC stimulation induces a stronger T cell response that lasts longer than the humoral response [37]. The latter two platforms have served as the foundation for Shenzhen Genoimmune Medical Institute vaccines [21], which are currently in clinical trials [38].

Table 2 shows the characteristics of the main anti-SARS-CoV-2 vaccines.

## 3. Various Vaccine Delivery (Administration) Methods

When analyzing the routes of administration of COVID-19 vaccines, the most common method is intramuscular, followed by intranasal, and finally intradermal (Table 3) [7].

Intranasal vaccine administration is a promising option. Because SARS-CoV-2 primarily affects the respiratory tract, including the lungs, inducing mucosal immunity is essential for protection. The formation of adaptive immunity at the site of antigen primary penetration (inductor site) without the inclusion of a draining lymph node or spleen is a feature of mucosal immune response development. The already-formed effector lymphocytes migrate to the mucosal effector site. After activation in the inductor site, mucosal T and B cells migrate through the lymph and blood to various effector sites, where they differentiate into Th effectors, cytotoxic CD8+ T lymphocytes, and plasma cells [56]. Mucosal immunity results in the production of specific IgA directly in the mucosa, as well as neutralizing IgG and a specific response of T cells, which reduces the replication of the SARS-CoV-2 virus in the nasal epithelium [57]. Nasal vaccines are being developed using vectors (adenovirus, Newcastle disease virus, influenza virus, parainfluenza, and respiratory syncytial virus), protein subunits, and live attenuated virus as platforms [58]. Nebulizers and pipettes are used to deliver the vaccine [41]. As of July 2022, three nasal vaccines were in phase 3 clinical trials: DelNS1-2019-nCoV-RBD-OPT1, СOVI-VAC, and BBV154 (Вharat Biotech International Limited, India (BBV154)) [7]. In 2022, Russia registered two nasal combination, two-component vector vaccines based on adenovirus types 26 and 5: Sputnik V [59] and Salnavac (JSC Generium, Russia (Salnavac)) [60].

The use of oral vaccines against COVID-19 is being considered; their advantage is that no injections are required; thus, no pain syndrome exists [61]. There are no registered oral vaccines on the market at the moment. A vector vaccine based on adenovirus type 5, VXA-CoV2-1 (Vaxart Inc, USA (VXA-CoV2-1)), which acts on spike (S) protein and nucleoprotein (NP) (N), is currently in a phase 2 trial [7,62,63]. Another oral vaccine, OraPro-COVID-19™ (iosBio Pharma, UK (OraPro-COVID-19™)), based on a non-replicating adenovirus, is also currently in clinical trials [64].

## 4. Monoclonal Antibodies for the Prevention of COVID-19

Anti-SARS-CoV-2 monoclonal antibodies are widely used for the treatment of COVID-19 infection, with moderate to high efficacy against both SARS-CoV-2 initial lines (B.1.351, P1, and B.1.1.7) and the Omicron variant [21]. However, the FDA has approved several products containing a combination of anti-SARS-CoV-2 monoclonal antibodies (casirivimab and imdevimab) for COVID-19 pre-exposure prophylaxis [65,66].

## 5. Immunogenicity and Safety of Vaccines

Vaccine effectiveness is determined by the induction of both humoral and cellular immune responses. As a result, quantifying neutralizing antibodies and the activity of specific T cells is recommended to assess vaccine efficacy and post-vaccination immunity [56,67,68]. According to mathematical modeling, post-vaccination neutralizing antibody titers are linearly related to vaccine efficacy [69]. The level of expression of each SARS-CoV-2 protein correlates with the prevalence and extent of CD4+ T cell responses to SARS-CoV-2 [70].

The ELISPOT platform is used by many researchers to assess the response of T cells induced by vaccination. T cell immune response was formed as early as 10 days after Sputnik Light vaccination, according to ELISPOT technology [54]. The ELISPOT-based Tigra Test SARS-CoV-2 kit detected T cell immune response 90 and 120 days after Sputnik V immunization, including in seronegative individuals [71].

The efficacy of the main vaccines against the Omicron strain is of interest in light of the ongoing mutation of the SARS-CoV-2 virus [72]. Omicron (B.1.1.529) avoids many of the neutralizing antibody responses elicited by vaccines due to multiple mutations. In contrast to humoral immunity, vaccine-induced cellular immunity is highly reactive against the Omicron strain [73].

Immunogenicity can have a direct impact on vaccine safety and tolerability. In clinical trials, the incidence of adverse events was significantly higher after mRNA and vector vaccine administration (local reactions in 40–89% of cases and systemic reactions in 44–86% of cases) than after inactivated vaccine administration (injection-site reactions in 5–23% of cases and systemic reactions in 4–18% of cases) [74].

According to a meta-analysis, adverse events such as fever are more common after receiving inactivated vaccines, while headache and muscle pain are more common after receiving adenovirus vector-based vaccines, and 56% of people who received mRNA vaccines reported fatigue [75].

When the incidence of serious adverse events associated with BNT162b2, Ad26.COV2.S, and mRNA-1273 vaccinations was examined, the BNT162b2 vaccine had the highest reported incidence of myocarditis. The Ad26.COV2.S vaccine was more frequently linked to the development of Guillain–Barré syndrome. Thrombotic complications occurred more frequently with BNT162b2 and Ad26.COV2.S vaccination than with mRNA-1273 vaccination [43].

## 6. Vaccinations of Various Populations

### 6.1. General Population

COVID-19 mRNA vaccines (BNT162b2 and mRNA-1273) are recommended by the US Centers for Disease Control and Prevention (CDC) for primary and booster vaccinations in all populations. Currently, primary vaccination with one type of vaccine is recommended [76]. The WHO considers the following vaccines to be safe and effective: AZD1222; Ad26.COV2.S; mRNA-1273; BNT162b2; BBIBP-CorV; COVID-19 Vaccine (Vero Cell), Inactivated; BBV152; and NVX-CoV2373 (all trade names). In a clinical setting, these vaccines are equivalent, and healthcare professionals should use whichever option is available [55]. In patients under 60 years old, Australian guidelines prefer BNT162b2, mRNA-1273, or Novavax vaccines over AZD1222 [77]. As of July 2022, ten vaccines had been approved for use in Russia. A long-acting combination monoclonal antibody (tixagevimab + cilgavimab) can be used for pre-exposure prophylaxis of COVID-19 in adults and children (aged 12 years and older weighing at least 40 kg) who are not infected with SARS-CoV-2, have not been in contact with a person infected with SARS-CoV-2, and have contraindications for vaccination against COVID-19 [78].

### 6.2. Use in Pregnancy

The CDC and the WHO recommend any approved COVID-19 vaccination for those pregnant, breastfeeding, trying to get pregnant now, or planning to get pregnant in the future [55]. Russian national guidelines recommend Sputnik V vaccination for pregnant women if the benefit outweighs the risk. Vaccination is contraindicated while breastfeeding [79].

### 6.3. Children and Adolescents

The CDC recommends the COVID-19 vaccination for anyone aged 6 months and older. BNT162b2 and mRNA-1273 are approved for use in children [76]. The WHO recommends starting the BNT162b2 vaccine at the age of five, and the mRNA-1273 vaccine at the age of twelve [62]. Vaccination is recommended for everyone over the age of five in Australia, [63] and over the age of twelve in Canada [80]. In Russia, the Gam-COVID-Vac-M vaccine is administered to children aged 12 to 17 [78].

### 6.4. People Who Have Had COVID-19

The CDC recommends vaccination for all people over the age of six months, regardless of whether they have a history of symptomatic or asymptomatic SARS-CoV-2 infection [76]. The WHO also supports this strategy [55]. In Australia, vaccination is recommended no sooner than three months after infection confirmation [63]. In Russia, vaccination is recommended 6 months after the disease, based on epidemic indications (including in previously vaccinated individuals) [81].

### 6.5. Immunocompromised Patients

For immunocompromised patients, the CDC recommends COVID-19 mRNA vaccines (BNT162b2 or mRNA-1273) for primary and booster vaccinations [76]. In this population, the WHO and Australian guidelines recommend additional doses of the BNT162b2 or mRNA-1273 vaccine [55,77]. Given the risk of more severe infection, Canadian guidelines recommend vaccinating patients with primary immunodeficiency with mRNA vaccines [82]. In Russia, the Sputnik V vaccine is recommended in this population. People with immunodeficiency who have had COVID-19 may receive Sputnik Light [81].

### 6.6. Booster Vaccination

Booster vaccination and revaccination (seasonal vaccination) against COVID-19 are used to maintain active immunity against COVID-19 for a longer period of time after primary immunization. A booster is the administration of a vaccine with the same or a different antigenic composition before the previous vaccine’s immunogenic effect has worn off. The next booster dose of the vaccine should increase the intensity and duration of post-vaccination immunity. The timing of the booster vaccination ranges from three to six months, depending on the country’s legislative framework [77,81,82,83,84]. Revaccination (seasonal vaccination) against COVID-19 is performed 12 months after the main (primary) or booster vaccination [85].

As recently as late 2021, the need for booster doses of COVID-19 vaccines was debatable [6]. Lately, public opinion on vaccine booster administration has shifted in favor of mandatory administration of booster doses, as this increases immunity duration and resistance to the Omicron strain [40,86]. The CDC recommends a booster injection for patients over the age of 12, with the first booster being administered three months after primary immunization. In patients over the age of 50, the second booster should be administered four months after the first [76]. According to WHO recommendations, one booster dose is sufficient [55]. In Australia, a single dose of a COVID-19 vaccine booster is recommended for people aged 16 and older who have completed their primary course three months ago or more. The second booster is recommended for people over the age of 50, nursing-home residents, people with severe immunosuppression, people with significant disabilities, and people at risk of severe COVID-19 infection [77]. In Canada, booster doses are recommended from 18 years of age [82]. In the United Kingdom, booster immunization with BNT162b2 or mRNA-1273 is currently recommended three months after primary vaccination [84]. In Russia, vaccination with Sputnik Light, EpiVacCorona, and AURORA-CoV is recommended [81].

Some vaccines have only been approved for use as boosters. Only a booster dose of the BNT162b2 vaccine is recommended for adolescents aged 12 to 17 years [87]. In August 2022, the mRNA-1273.214/Moderna^TX^ vaccine was registered (Moderna, USA (mRNA-1273.214)). This is a bivalent mRNA vaccine containing sequences encoding SARS-CoV-2 and Omicron variant spike proteins [88]. A systematic review as of May 2022 found that three doses of RNA vaccines provided the best efficacy for vaccination and booster vaccination (96% vaccine efficacy; 95% CI, 72% to 99%). The combination of two adenovirus vector vaccines and one RNA vaccine has an efficiency of 88% (range: 59–97%). The homologous two-dose regimen of RNA vaccines results in 99% (79% to 100%) vaccine efficacy in preventing severe COVID-19. Triple-dose RNA vaccines are the most effective in reducing COVID-19-related hospitalizations (95% CI, 90% to 97%). Three-dose regimens of homologous and heterologous strains have been shown to be effective in preventing infection with the Alpha, Delta, and Omicron strains [89].

## 7. Vaccines and New Strains

The WHO has currently registered six SARS-CoV-2 variants of concern. Three pre-existing variants—Alpha, Beta, and Gamma—have already lost their relevance. Delta and Omicron are the two most important variants right now [90].

The SARS-CoV-2 Alpha variant includes viral strains with 23 mutations, including 7 mutations and 2 deletions in the RNA fragments encoding the S protein [91]. This variant is significantly more virulent, with a higher risk of death or severe COVID-19. The Beta variant mutations include nine mutations and one deletion in spike (S) protein [92]. The variant is distinguished by a significant acceleration of disease spread when compared with the reference strain [93]. In the Gamma variant of SARS-CoV-2, 12 mutations alter the structure of the S protein, increasing virulence. In the Delta variant, there are 19 known mutations in the S protein that alter the structure of the RBD of the S protein [91]. The Omicron strain has a high ability to mutate, resulting in the formation of numerous sublines, and new variants have significant genomic differences from the first lines [93]. The main problems with Omicron are that it is more infectious or dangerous than other variants of concern and that it can bypass vaccine-induced immunity. When tested in patients’ plasma after triple vaccination and subsequent infection with BA.1, subspecies BA.2.12.1, BA.4, and BA.5 showed higher transmissibility and lower antibody neutralizing ability than BA.2 [94]. The most recent SARS-CoV-2 mutations from the strain BA.2.75 subspecies, nicknamed “Centaurus” and “Pisces”, are thought to be more resistant to vaccine-induced immunity than previous strains [90,95].

The WHO recommends a vaccine efficacy threshold of 50% [96].

Neutralizing antibodies induced by BNT162b2 and mRNA-1273 are lower than those induced by P.1 and B.1.351. The Ad26.COV2.S vaccine was 64.0% effective in the Brazilian population and 52% effective in the South African population against variant B.1.351; NVX-CoV2373 was shown to be 49% effective in the South African population. The BNT162b2 vaccine was found to be effective against the P.1 variant. The efficacy of BNT162b2, mRNA-1273, AZD1222, and COVID-19 Vaccine (Vero Cell), Inactivated, against Gamma and Delta variants was estimated as 85%, 78%, 70%, and 66%, respectively. Two doses of the BNT162b2 vaccine were found to cross-neutralize some circulating Delta variants, while the Ad26.COV2.S vaccine’s efficacy against this virus variant was reduced from 66.9% to 60%. The mRNA-1273 vaccine was found to be approximately 94.1% effective against the Delta variant when compared with the BNT162b2 and Ad26.COV2.S vaccines, while the BNT162b2 and AZD1222 vaccines were less effective [97]. In an Indian study, BBV152 and AZD1222 were 93% and 94% effective, respectively, against the Delta strain [98]. The Sputnik V vaccine was 81% effective in preventing the hospitalization of patients with the Delta variant in preclinical studies [99].

BNT162b2 (35%), mRNA-1273 (20% after the first dose, 42.8% after the second dose, 67.7% after the third dose), Ad26.COV 2.S (47% after two doses, 63% when given as a booster), and AZD1222 (11.44–51%) all demonstrated activity against the Omicron strain [100,101]. Monoclonal antibodies are also active against Omicron [42].

The serum titer of neutralizing antibodies to the Omicron variant in patients revaccinated with Sputnik Light was statistically comparable to the serum titer of neutralizing antibodies to the B.1.1.1 variant in patients vaccinated with Sputnik V. Despite a decrease in titer, neutralizing antibodies to Omicron were detected in the serum of 100% of revaccinated individuals after Sputnik Light revaccination [49].

In Canada, mRNA-1273.214/Spikevax Bivalent Original/Omicron BA.1 (Moderna, USA (mRNA-1273.214)), containing mRNA encoding the original SARS-CoV-2 virus and Omicron BA.1, has been approved for use as a booster. When compared with mRNA-1273 as a second booster dose, this vaccine induces higher neutralizing antibody titers against the parent strain, as well as Omicron BA.1, Omicron BA.4, and Omicron BA.5 [102]. mRNA-1273.222/Spikevax Bivalent Original/Omicron BA.4/BA.5 (Moderna, USA (mRNA-1273.214)) has been approved in the USA [103].

Pfizer-BioNTech has launched two bivalent vaccines: BNT162b2 (B.1.1.529)/BNT162b2 Bivalent/Comirnaty Bivalent Original/Omicron BA.1, produced by Pfizer/BioNTech, USA (BNT162b2 (B.1.1.529)); and BNT162b2 Bivalent BA.4/BA.5/Comirnaty Bivalent Original/Omicron BA.4/BA.5, produced by Pfizer-BioNTech, USA (BNT162b2 Bivalent). Both vaccines contained one dose of Comirnaty and one dose of Omicron subvariant vaccine (BA.1 or BA.4/BA.5) [104].

Phase 3 studies are ongoing for Omicron COVID-19 Inactivated Vaccine (Vero Cell), produced by China National Biotec Group Company Limited, China (Omicron COVID-19 Inactivated Vaccine (Vero Cell)), an inactivated virus vaccine [7,105].

## 8. Immunization Schedule

The CDC has developed a COVID-19 vaccination schedule for children starting at 6 months of age, in which a booster is administered no later than 5 months after two primary doses of one vaccine, and people over 50 years of age should receive a second booster after 4 months [68].

In Belarus, revaccination (seasonal vaccination) with Sputnik V, Sputnik Light, BBIBP-CorV, or another immunobiological drug is recommended 12 months after the booster vaccination for persons 18 years of age and older and after the main (primary) vaccination for those who did not receive booster vaccination, during periods of seasonal increase in the incidence [85].

In Russia, citizens in three priority levels are subject to COVID-19 vaccination based on epidemic indications. The first level includes people aged 60 and older who work in service organizations, have chronic diseases, and live in large cities. The second level includes civil servants and employees of law enforcement and other bodies, as well as service workers. The third level includes children aged 12 to 17, pupils and students, army conscripts, and office workers [106].

## 9. Conclusions

The updated review reveals that, despite all efforts, the SARS-CoV-2 virus has not yet been eradicated. The virus’s ability to mutate indefinitely has resulted in the emergence of new strains that are resistant to previously acquired immunity and can bypass previously formed immune mechanisms.

This necessitates the development of new, more effective vaccines. The vaccine market is rapidly expanding, with new products appearing all the time. A total of 42 vaccines have already been approved for use. To date, the top three most influential COVID-19 vaccine platforms have not changed, with mRNA, VVnr, and inactivated virus vaccines maintaining their positions. However, focus has shifted to PS vaccines.

The development of COVID-19 vaccination has increased not only the availability of vaccines, but also the ease with which they can be used. For example, single-dose vaccines and nasal vaccines are already in use, and oral vaccines are being developed, which will undoubtedly simplify their administration.

New products, including those based on the inactivated Omicron virus, are being developed to improve the efficacy of vaccines against new strains of SARS-CoV-2. The approach to booster doses and revaccination has also been reconsidered. Currently, the use of one or even two booster vaccines is recommended, which does not eliminate the need for revaccination.

With the accumulation of experience in the use of COVID-19 vaccines, the number of people who can be vaccinated has increased while the list of contraindications has decreased. Vaccines are now known to be safe for children, pregnant women, the elderly, and people with immunosuppressive conditions. Vaccination is recommended even for people who have already had COVID-19.

The fast-paced process of combating SARS-CoV-2 is associated with the constant updating of available information, as well as the change of guidelines, which necessitates regular review as new changes emerge.

## Figures and Tables

**Table 1:** Countries with the most vaccine research and/or the greatest numbers of vaccine registrations [8].

Country	Vaccine Trials	Approved Vaccines	Vaccines
Argentina	18	9	mRNA-1273, BNT162b2, Ad5-nCoV, Sputnik Light, Sputnik V, AZD1222 (Vaxzevria + Covishield), BBIBP-CorV, and COVID-19 Vaccine (Vero Cell)
Australia	31	5	NVX-CoV2373, mRNA-1273, mRNA-1273.214, BNT162b2, Ad26.COV2.S, and AZD1222
Belgium	19	6	NVX-CoV2373, mRNA-1273, mRNA-1273.214, BNT162b2, BNT162b2 (B.1.1.529), BNT162b2 Bivalent, Ad26.COV2.S, AZD1222, and VLA2001
Brazil	32	7	BNT162b2, Sputnik V, Ad26.COV2.S, AZD1222 (Vaxzevria + Covishield), BBIBP-CorV, and COVID-19 Vaccine (Vero Cell)
Canada	25	8	NVX-CoV2373, Covifenz, mRNA-1273, mRNA-1273.214, BNT162b2, Ad26.COV2.S, and AZD1222 (Vaxzevria + Covishield)
China	98	8	Zifivax, V-01, Ad5-nCoV-IH, Ad5-nCoV, KCONVAC, BBIBP-CorV, and COVID-19 Vaccine (Vero Cell) (Inactivated (Vero Cells) + CoronaVac)
Germany	31	9	NVX-CoV2373, mRNA-1273, mRNA-1273.214, BNT162b2 (B.1.1.529), BNT162b2 Bivalent, Ad26.COV2.S, AZD1222, and VLA2001
India	36	12	NVX-COV2373, Corbevax, ZyCoV-D, GEMCOVAC-19, mRNA-1273, iNCOVACC, Sputnik Light, Sputnik V, Ad26.COV2.S, AZD1222 (Vaxzevria + Covishield), and BBV152
Indonesia	22	13	Zifivax, IndoVac, NVX-COV2373, mRNA-1273, BNT162b2, AWcorna, Ad5-nCoV, Sputnik V, Ad26.COV2.S, AZD1222, KCONVAC, BBIBP-CorV, and COVID-19 Vaccine (Vero Cell)
Islamic Republic of Iran	26	12	Noora vaccine, Soberana 02, Razi Cov Pars, SpikoGen, Sputnik Light, Sputnik V, Ad26.COV2.S, AZD1222, BBV152, FAKHRAVAC (MIVAC), COVIran Barekat, and BBIBP-CorV
Japan	44	7	TAK-019, mRNA-1273.214, BNT162b2, BNT162b2 (B.1.1.529), BNT162b2 Bivalent, TAK-919, and AZD1222
Philippines	22	11	NVX-CoV2373, BNT162b2, mRNA-1273, Sputnik Light, Sputnik V, Ad26.COV2.S, AZD1222, BBV152, BBIBP-CorV, and COVID-19 Vaccine (Vero Cell) (Inactivated (Vero Cells) + CoronaVac)
Republic of Korea	22	8	NVX-CoV2373, SKYCovione, mRNA-1273, mRNA-1273.214, BNT162b2, BNT162b2 (B.1.1.529), Ad26.COV2.S, and AZD1222
Russia	33	6	Aurora-CoV, EpiVacCorona, Gam-COVID-Vac, Sputnik Light, Sputnik V, and KoviVac
South Africa	26	6	NVX-COV2373, BNT162b2, Ad26.COV2.S, AZD1222, BBIBP-CorV, and COVID-19 Vaccine (Vero Cell)
Spain	22	9	NVX-CoV2373, mRNA-1273, mRNA-1273.214, BNT162b2, BNT162b2 (B.1.1.529), BNT162b2 Bivalent, Ad26.COV2.S, AZD1222, and VLA2001
Thailand	22	7	NVX-COV2373, mRNA-1273, BNT162b2, Ad26.COV2.S, AZD1222, BBIBP-CorV, and COVID-19 Vaccine (Vero Cell)
United Kingdom of Great Britain and Northern Ireland	29	8	NVX-CoV2373, mRNA-1273, mRNA-1273.214, BNT162b2, BNT162b2 (B.1.1.529), Ad26.COV2.S, AZD1222, and VLA2001
United States of America	109	6	NVX-CoV2373, mRNA-1273, mRNA-1273.214, BNT162b2 Bivalent, and Ad26.COV2.S

**Table 2:** Characteristics of the main anti-SARS-CoV-2 vaccines.

Vaccine	Type	Dose Regimen	Prevention of Symptomatic Infection, % (95% CI)	Efficacy against the Omicron Strain	Main Adverse Events
BNT162b2	mRNA	2 doses, 3-week interval	95.0 (90.3–97.6) [39]	65.5% (95% CI, 63.9–67.0), 2–4 weeks after two doses; 8.8% (95% CI, 7.0–10.5), ≥25 weeks after two doses [40]	Myalgia, arthralgia, pain in the extremities, nervous system disorder, and headache [41]
mRNA-1273	mRNA	2 doses, 4-week interval	93.2 (91.0–94.8) [39]	20% after the first dose, 42.8% after the second dose, 67.7% after the third dose [42]	Thrombotic complications [43]
Ad26.COV2.S	VV	Single dose	66.5 (55.5–75.1) [44]		Guillain–Barré syndrome and thrombotic complications [43]
AZD1222	VV	2 doses, 4-week interval	67.1 (52.3–77.3) [45]	When assessed five months after the second dose, there was no protective effect against symptomatic Omicron infection after two doses [46]	Disseminated intravascular coagulation, thromboembolic events, injection-site pain, erythema, myalgia, arthralgia, and headache [47]
Sputnik V	VV	2 doses, 3-week interval	91.1 (83.8–95.1) [48]	The serum titer of neutralizing antibodies to the Omicron variant in patients re-vaccinated with Sputnik Light was statistically comparable to the serum titer of neutralizing antibodies to the B.1.1.1 variant in patients vaccinated with Sputnik V [49]	
Sputnik Light	VV	1 dose			Any injection-site symptoms, injection-site pain, erythema, general symptoms, flu-like syndrome, fatigue, headache, muscle and joint pain, pyrexia, chills, decreased appetite, rash, and dizziness [50]
BBIBP-CorV	IV	2 doses, 4-week interval	78.1 (64.9–86.3) [51]		Injection-site redness and swelling, and fever [52]
CoronaVac	IV	2 doses, 4-week interval	83.5 (65.4–92.1) [53]		Pain, erythema, and swelling at the injection-site, as well as weakness, myalgia, nausea, and chills [53]
Ad5-nCoV	VV	1 dose	58 [54]		Thrombosis with thrombocytopenia syndrome, Guillain–Barré syndrome, and anaphylaxis [54]
NVX-CoV2373	PS				
BBV152	IV	2 doses, 4-week interval [55]			

**Table 3:** Variants of vaccine delivery (administration) methods [7].

Route of Administration	Number	Main Variants
Oral	5	VXA-CoV2-1 Ad5 adjuvanted Oral Vaccine platform, bacTRL-Spike oral DNA vaccine, CoV2-OGEN1, protein-based vaccine, and COVID19 Oral Vaccine Consisting of Bacillus Subtilis Spores
Sub cutaneous	5	IMP CoVac-1, COVID-19/aAPC vaccine, hAd5 S+N bivalent vaccine, SARS-CoV-2-RBD-Fc fusion protein (AKS-452), and SARS-CoV-2 VLP Vaccine
Intradermal	9	INO-4800+electroporation, nCov vaccine, CORVax12-Spike (S) Protein Plasmid DNA Vaccine, GLS-5310, COVIGEN, EXG-5003, Plasmid DNA vaccine, PepGNP-SARSCoV2, Ad26.cov2.s + bcg vaccine, and XS-1223U
Intramuscular	142	BNT162b2, mRNA-1273, Ad26.COV2.S, AZD1222, Sputnik V, Sputnik Light, BBIBP-CorV, CoronaVac, Ad5-nCoV, and BBV152
Intranasal	13	Sputnik V, DelNS1-2019-nCoV-RBD-OPT1, COVI-VAC, CIGB-669, AdCOVID, BBV154, MV-014-212, PIV5, NDV-HXP-S, ACM-SARS-CoV-2-beta ACM-CpG, and Salnavac
Aerosol	1	Ad5-triCoV/Mac
Inhaled	2	MVA-SARS-2-ST Vaccine
